# Early Warning Software for Emergency Department Crowding

**DOI:** 10.1007/s10916-023-01958-9

**Published:** 2023-05-26

**Authors:** Jalmari Tuominen, Teemu Koivistoinen, Juho Kanniainen, Niku Oksala, Ari Palomäki, Antti Roine

**Affiliations:** 1 Faculty of Medicine and Health Technology, Tampere University, Tampere, Finland; 2 Faculty of Information Technology and Communication Sciences, Tampere University, Tampere, Finland; 3 Centre for Vascular Surgery and Interventional Radiology, Tampere University Hospital, Tampere, Finland and Finnish Cardiovascular Research Center, Tampere, Finland; 4 Kanta-Häme Central Hospital, Hämeenlinna, Finland

**Keywords:** Emergency department, Crowding, Overcrowding, Forecasting, Prospective, Software, ETS models

## Abstract

Emergency department (ED) crowding is a well-recognized threat to patient safety and it has been repeatedly associated with increased mortality. Accurate forecasts of future service demand could lead to better resource management and has the potential to improve treatment outcomes. This logic has motivated an increasing number of research articles but there has been little to no effort to move these findings from theory to practice. In this article, we present first results of a prospective crowding early warning software, that was integrated to hospital databases to create real-time predictions every hour over the course of 5 months in a Nordic combined ED using Holt-Winters’ seasonal methods. We show that the software could predict next hour crowding with an AUC of 0.94 (95% CI: 0.91-0.97) and 24 hour crowding with an AUC of 0.79 (95% CI: 0.74-0.84) using simple statistical models. Moreover, we suggest that afternoon crowding can be predicted at 1 p.m. with an AUC of 0.84 (95% CI: 0.74-0.91).

## Introduction

Emergency department (ED) crowding is a well-recognized threat to patient safety and it has been repeatedly associated with increased mortality [1–5]. Crowding is both a chronic and an international issue but despite a multitude of studies and media coverage, the problem seems to be getting worse [6]. According to a conceptual model, causes of ED crowding can be divided into three high-level components: input, throughput and output [7]. The importance and persisting relevance of this model was highlighted in a recent review article by *Morley et al 2018*, in which three respective phenomena were identified as the most important underlying causes for crowding: 1) increased number of patients with more urgent and complex care needs, 2) nursing staff shortages and 3) access block (i.e. difficulty to move patients from the ED to follow-up care after initial assessment and immediate treatment) [8]. From the point of view of an individual ED administrator, it is difficult to influence these factors, since majority of the required interventions are locked behind slow political processes scattered throughout the health care system.

For these reasons, there is a continued interest aiming to enhance the use of the limited resources that are readily available. One notable manifestation of this effort is emergency department forecasting, which has established itself as a small but persistent research niche [9]. The rationale of the forecasting work is simple: 1) forecast future service demand, 2) enable proactive administrative decisions, 3) ensure sufficient resources and 4) improve treatment outcomes. Despite many recent advancements in forecasting methodology [10–13], several gaps in the literature remain.

First, there is little to no knowledge about the performance of the forecasting models in predicting future crowding in binary terms. This is because models have been predominantly assessed using continuous error metrics such as mean absolute percentage error (MAPE) or root mean squared error (RMSE) [9], which are useful when models are compared between one another. However, these metrics are not transferrable between facilities and they are hard to communicate to ED administration, who are often more familiar with categorical metrics that are widely used in diagnostics. We thus argue that the performance of proposed forecasting models should be increasingly reported using discrete metrics that are easily interpretable by administrative stakeholders of the ED.

Categorization is also important when the association between crowding and patient outcomes is investigated. In fact, many of the studies that have documented an association between mortality and crowding have done so by comparing the most crowded quartile between less crowed ones [1–3]. Although the underlying association between increased occupancy and mortality is likely a continuous one, this kind of categorization is beneficial from practical standpoint, since decision-making is known to benefit from actionable, simple output [14]. We argue that the most relevant definition for crowding is mortality-associated crowding, which is increasingly defined in categorical terms. As such, categorical crowding should be the primary target variable of an ED forecasting system.

In Tampere University Hospital, following the rationale above, the ED is considered crowded when a certain occupancy threshold is exceeded. This is coupled with a catchment-area-wide protocol that aims to resolve the observed crowding. The protocol mandates the shift-supervising physician to call-in additional staff and obligates follow-up care facilities to accept patients even if their nominal capacity has been exceeded. The obvious problem with this approach is the delay between actions and outcomes, which leads to prolonged crowded state, increased length of stay and decreased quality of care. Our ultimate goal is to move these administrative manoeuvres from reactive space into proactive space by offering accurate forecasts about the future status of the ED.

Second, vast majority of previous work has been based on historical simulations [9]. A prominent exception to this rule was offered by *Hoot et al 2009* in which a prospective ED forecasting system *ForecastED* was presented and validated with promising results[15]. Ever since, the interest towards conducting prospective studies has for some reason withered. This is unfortunate, because although retrospective studies are obviously useful in identifying potential forecasting models their impact will remain limited unless the findings are confirmed prospectively in real-life setting. We believe it is time to return to prospective evaluation and aim to offer an easily interpretable baseline to compare more advanced models against.

Third, ED forecasting models have been under examined in the Nordic countries with few notable exceptions [16, 17]. We also note that increasingly complex solutions are reported retrospectively [10–12] but since they are only evaluated in continuous terms, it is difficult to assess whether these models have practical utility. Meanwhile, even the most rudimentary statistical models remain unassessed in discrete terms.

In this study, we aim to fill the gaps identified above. We developed an early warning software that was integrated to Tampere University hospital databases to make hourly predictions of ED arrivals and occupancy 24 hours ahead using established statistical models. In this study, we report the performance of the system in predicting discrete future crowding along with its reliability.

## Materials and Methods

### Study Setting

Tampere University Hospital is an academic hospital located in Tampere, Finland. It serves a population of 535,000 in the Pirkanmaa Hospital District and, as a tertiary hospital, an additional population of 365,700, providing level 1 trauma centre capabilities. The hospital ED, *Acuta*, is a combined ED with a nominal capacity of 106 patients, with 65 beds and 41 seats for walk-in patients. Approximately 90,000 patients are treated annually making *Acuta* one of the largest EDs in Scandinavia.

For the purposes of this study, we developed a forecasting software that had following requirements: 1) it had to be able to forecast both future arrivals and occupancy, 2) it had to operate with hourly data, making predictions 24 hours ahead, 3) the predictions had to be stored in a database for later accuracy evaluation and 4) it had to have a rudimentary user interface for debugging purposes. The software was deployed to an Azure cloud computing service (Microsoft Corporation, USA) on January 15, 2022 and predictions were made until May 26, 2022 constituting a total of 3145 hours. To ensure safety of sensitive patient information the virtual machine was isolated from hospital databases and only the high-level statistics required to make predictions were provided to a dedicated database.

### Definition of Crowding

In this study, our main goal is to assess model performance in predicting binary crowding. Unfortunately, international and commonly accepted standard definition for crowding does not exist. The proposed crowding metrics such as NEDOCS [18] or EDWIN [19] are too complex to be used here and they are difficult to apply to a Nordic combined ED. For these reasons we will define the most crowded quartile of days to be crowded similiar to the way was done by *Richardson et al 2006* [1] with most crowded shifts. This is done using a proxy metric called *daily peak occupancy (DPO)* which is defined as the highest recorded occupancy of the day.

### Models

The software included three forecasting models: Holt-Winter’s additive method (AHWM), Holt-Winters’ multiplicative method (MHWM) and Holt-Winters’ damped method (DHWM) [20]. These models were selected due to their established status, capability to process seasonal data and efficiency in terms of computing power. The models, later referred collectively to as ETS models (E stands for error term, T for trend component, and S for seasonal component) were trained with all available historical data and implemented with Statsmodels Python module [21]. ETS model parameters were determined using maximum likelihood estimation.

### Performance Metrics

The performance of these algorithms are reported in four distinct phases: (1) aggregated continuous performance, (2) performance per horizon (3) performance per origin and (4) performance with pairwise origin and horizon combinations. Only out-of-sample performance results (i.e. results obtained with test data) are presented in this study. In the first phase, the continuous performance of the models is reported using mean absolute error (MAE) and root mean squared error (RMSE). The errors are averaged over all the forecast horizons.

In phases 2-4, we evaluate the performance of the models as binary predictors of future crowding. Area under the receiver operating characteristics curve (AUROC/AUC) were used as the main error metric in both phases and detailed unadjusted binary metrics, such as F1, are provided in the Appendix. F1 is an unweighted harmonic mean of precision and sensitivity (recall), which is often recommended as a performance measure under class imbalance [22]. Due to class-imbalance between crowded and noncrowded states, the noncrowded states were randomly downsampled to match the number of crowded ones. This was done to eliminate potential positive bias in AUC values. 95% confidence intervals (CI) were calculated for AUC values using bootrap method with 250 iterations [23].

*Performance per horizon (PPH)* is documented in phase two. This allows the reader to assess how well the model is able to predict whether the ED will be crowded exactly $t$ steps ahead. For example, $t+2$ PPH for predictions made at 1 p.m. tells whether the ED will be crowded between 3 p.m. and 4 p.m. Each horizon from 1 to 24 hours are independently assessed. The issue with PPH metric is that the results are aggregated over different forecast origins although performance can vary significantly depending on the said origin.

*Performance per origin (PPO)* in phase three resolve this issue. In this phase we assess whether the models are able to tell whether the next 24 hours will be overcrowded as the forecast origin moves from 0 a.m. to 23 p.m. We hypothesize that the accuracy is lowest at midnight and gradually increases throughout the day when the models is given new information about the status of the ED. The approach aims to simulate the way the forecasts would be used in practice if offered to ED administration. In this phase, the future is considered crowded if the occupancy reaches the highest quartile for one or more hours within the 24 hour forecast window.

*AUC matrix* is provided in phase four, showing pairwise AUC results for different origin and horizon combinations. This aims to further eliminate the confounding impact of changing forecast origin in aggregated PPH metrics.

## Results

### Descriptive Statistics

**Fig. 1:**
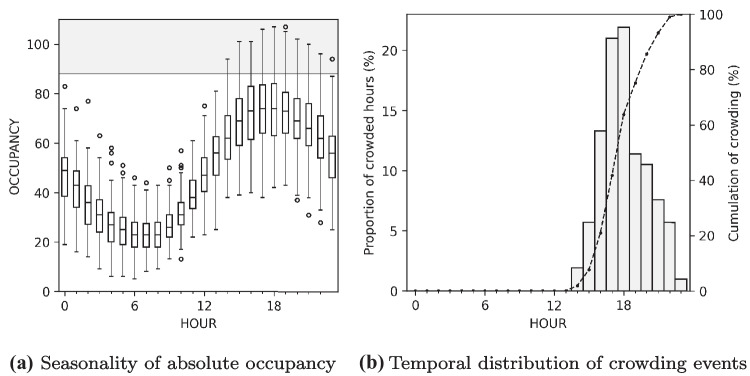
Hourly target variable seasonalities. The grey area shows the overcrowded state

Hourly target variables followed a clear seasonality. On average there were only 2 hourly arrivals between 5-7 a.m. with gradual increase over the course of the morning and throughout the day. Arrivals peaked at 4 p.m. with median of 13 patients and maximum of 23. Occupancy followed a similar sinusoidal shape with slight delay compared to arrivals. The occupancy was lowest between 6-8 a.m. with median of 23 patients and peaked between 5-6 pm. with median of 74 and maximum of 107 patients as shown in Fig. 1a.

Temporal distribution of crowding events, shown in Fig. 1b, was a direct result of the seasonality described above. On hourly resolution, all the crowding events were observed after 2 p.m. and most of them at 6 p.m. (22 %).

Distributions of the *daily peak occupancy* and *hourly occupancy* are provided in the Appendix “Occupancy Distributions”. Based on DPO statistics, the highest quartile was observed at occupancy of 88. This annotates, by definition, 25 % of days as crowded and 3 % of the highest occupancy hours as crowded.

The ED visit statistics were delivered reliably to the dedicated database and there were no missing data as regards to target variables. This was not the case with the predictions. There was a downtime of two weeks from February 14th to 27th and three days from April 12th to 14th due to a credentials issue. This resulted in total missing data of 397 hours (12%) for all of the models. In addition, due to an unresolved issue with memory management, the software experienced sporadic downtime resulting in missing data. This contributed to an additional modelwise data loss ranging between 82-108 ($\sim 3$ %). Temporal distribution of missing data is shown in Fig. 3 in Appendix.

**Fig. 2:**
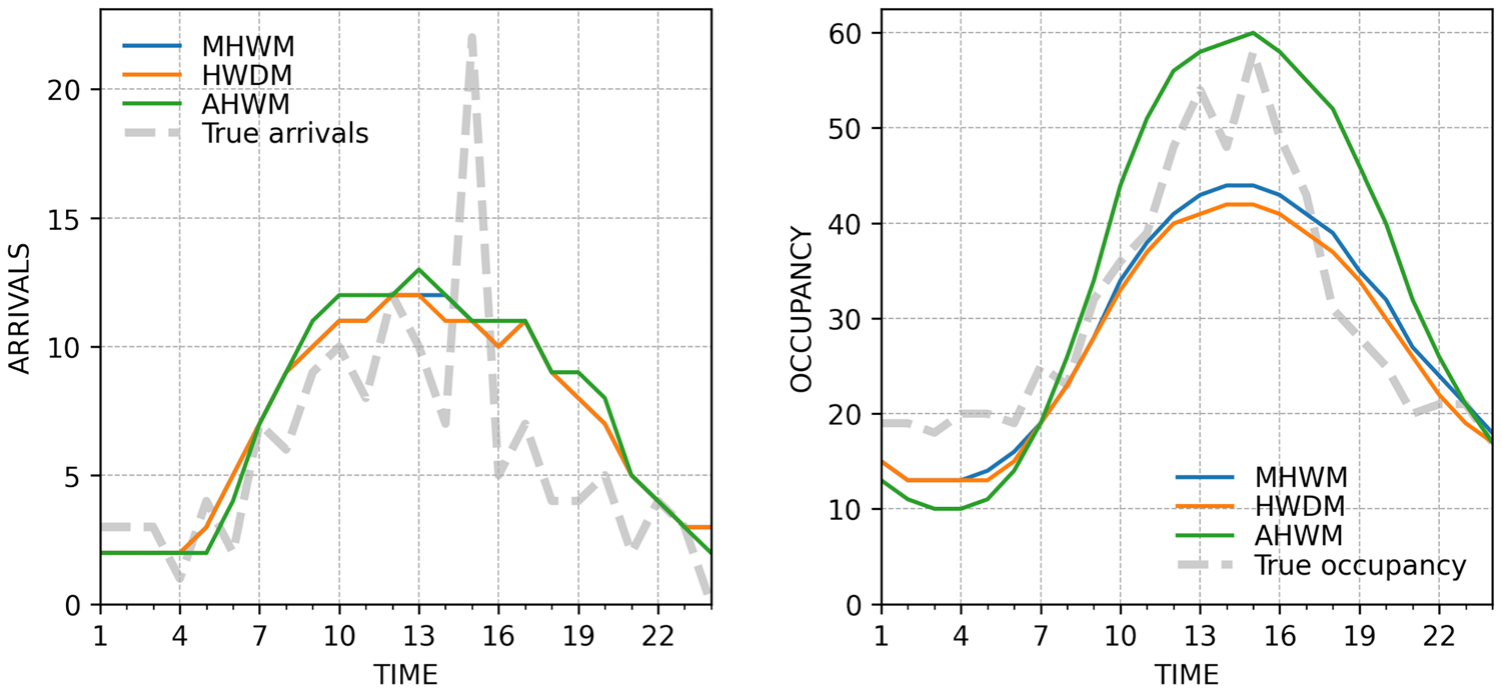
Example predictions on 2022, February 13th at 4 a.m. Note the almost identical performance when forecasting arrivals, which is also reflected in the overall accuracy results. In case of occupancy, AHWM is closest to the ground truth whereas other ETS models a clear negative bias

### Continuous Performance

Continuous accuracy metrics are provided in Table 1. AHWM was the most accurate model in terms of continuous occupancy with MAE of 10.22 and RMSE of 176.59. The RMSEs of HWDM and MHWM were 35% and 24% higher than that of AHWM likely because of their negative bias as demonstrated in Fig. 2, which unquestionable favours AHWM. In contrast, when it comes to the prediction of arrivals, the models were almost of equal performance, HWDM being slightly the most accurate with MAE of 2.10 and RMSE of 7.58. An example of the predictions is provided in Fig. 2.

**Table 1:** Continuous aggregated error metrics reported with Mean Absolute Error (MAE) and Root Mean Squared Error (RMSE)

		MAE	RMSE
Target	Model		
Occupancy	HWDM	11.69	273.47
	MHWM	10.87	233.38
	AHWM	10.22	176.59
Arrivals	AHWM	2.13	7.64
	MHWM	2.11	7.59
	HWDM	2.10	7.58

### Binary Performance

**Fig. 4:**
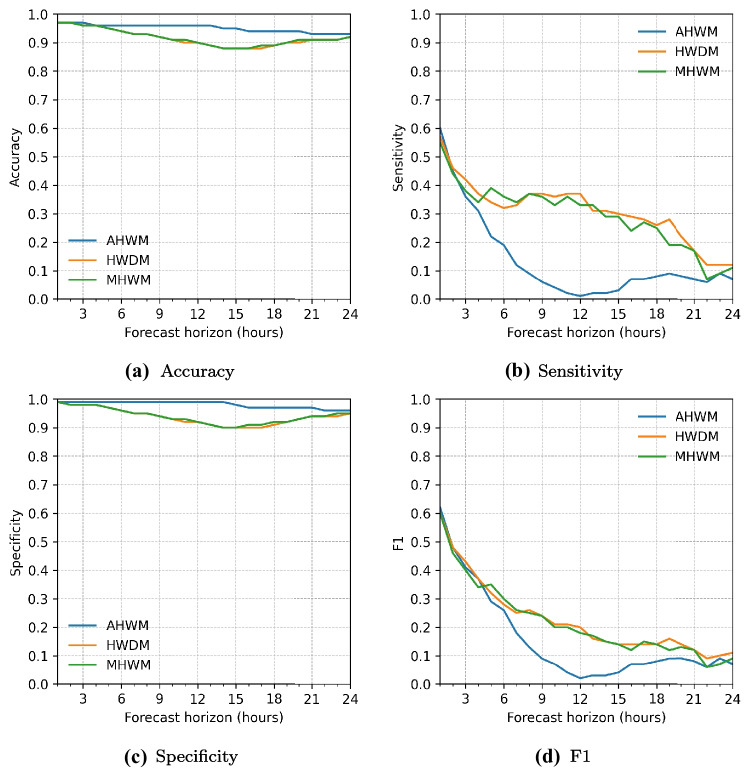
Performance as function of forecast horizon

**Fig. 5:**
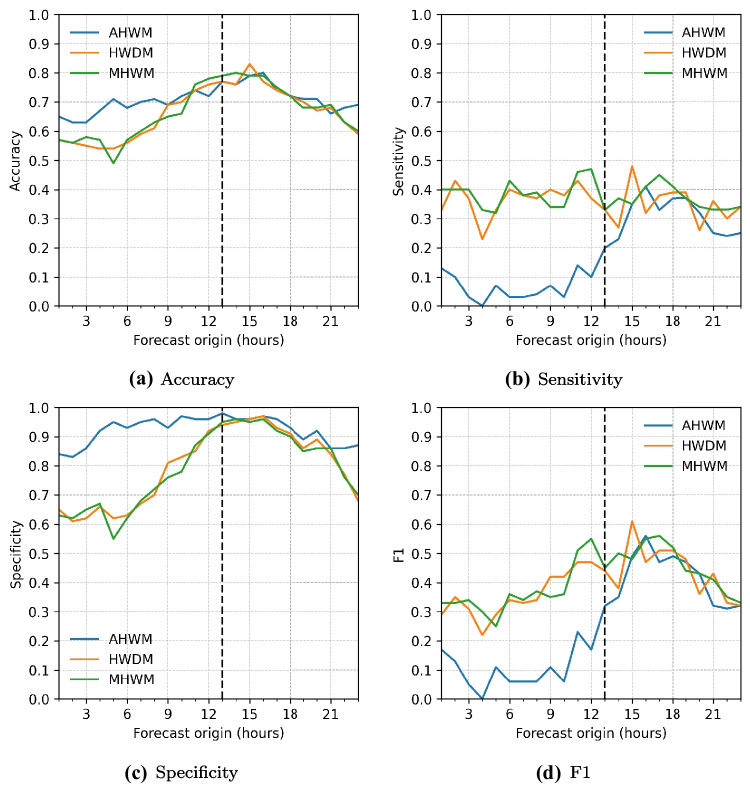
Performance as function of forecast origin

Binary performance results as measured by AUC are provided in Tables 2 and 3. In addition, unadjusted and more detailed metrics are provided in Figs. 4 and 5 in Appendix. The AUCs of one-step ahead PPH predictions were high 0.94 (95% CI: 0.91-0.96) for all the forecasting models. The accuracy of the ETS models decreased monotonically as a function of forecast horizon and bottomed at 0.79 (95% CI: 0.75-0.84) at $t+24$. PPO was low at 0 a.m. with AUC ranging between 0.50-0.52 but the performance increased gradually over the course of the day. HWDM reached an AUC 0.72 (95% CI: 0.60-0.82) at 11 a.m., 0.78 (95% CI: 0.67-0.87) at 12 a.m., 0.84 (95% CI: 0.74-0.91) at 1 p.m. and finally 0.88 (95% CI: 0.80-0.95) at 3 p.m. Differences between HWDM and other ETS models were small. AUC matrix showing results based on different origin and horizon combinations is provided in Table 4. Only AHWM results are provided here for brevity.

**Table 2:** The performance of the models over different forecast horizons (PPH) Area Under Curve (AUC). 95 % confidence interval in the parenthesis

Horizon	AHWM	HWDM	MHWM
t+1	0.94 (0.91-0.97)	0.94 (0.91-0.96)	0.94 (0.91-0.97)
t+2	0.93 (0.89-0.95)	0.93 (0.90-0.96)	0.92 (0.89-0.95)
t+3	0.91 (0.88-0.95)	0.90 (0.86-0.93)	0.89 (0.85-0.93)
t+4	0.90 (0.86-0.94)	0.89 (0.84-0.93)	0.90 (0.86-0.94)
t+5	0.90 (0.86-0.93)	0.88 (0.84-0.92)	0.90 (0.85-0.93)
t+6	0.90 (0.86-0.93)	0.86 (0.81-0.90)	0.89 (0.86-0.92)
t+7	0.90 (0.86-0.94)	0.85 (0.81-0.90)	0.86 (0.81-0.90)
t+8	0.89 (0.85-0.93)	0.85 (0.81-0.90)	0.85 (0.80-0.90)
t+9	0.86 (0.81-0.90)	0.87 (0.82-0.91)	0.88 (0.83-0.92)
t+10	0.90 (0.86-0.94)	0.86 (0.81-0.90)	0.86 (0.81-0.91)
t+11	0.82 (0.76-0.87)	0.85 (0.81-0.89)	0.81 (0.75-0.86)
t+12	0.84 (0.79-0.88)	0.84 (0.79-0.89)	0.82 (0.78-0.87)
t+13	0.83 (0.77-0.87)	0.83 (0.78-0.88)	0.82 (0.77-0.87)
t+14	0.82 (0.77-0.87)	0.80 (0.74-0.86)	0.78 (0.73-0.84)
t+15	0.81 (0.76-0.87)	0.78 (0.71-0.84)	0.77 (0.70-0.83)
t+16	0.80 (0.74-0.85)	0.76 (0.70-0.81)	0.76 (0.70-0.83)
t+17	0.79 (0.73-0.84)	0.76 (0.69-0.82)	0.77 (0.71-0.83)
t+18	0.78 (0.73-0.84)	0.73 (0.66-0.79)	0.77 (0.71-0.83)
t+19	0.79 (0.72-0.84)	0.76 (0.70-0.81)	0.76 (0.70-0.82)
t+20	0.79 (0.73-0.83)	0.75 (0.69-0.81)	0.76 (0.70-0.81)
t+21	0.78 (0.71-0.83)	0.75 (0.69-0.80)	0.80 (0.74-0.85)
t+22	0.79 (0.73-0.84)	0.74 (0.68-0.80)	0.82 (0.77-0.87)
t+23	0.78 (0.72-0.83)	0.74 (0.68-0.80)	0.79 (0.72-0.84)
t+24	0.79 (0.74-0.85)	0.75 (0.70-0.81)	0.79 (0.74-0.84)

**Table 3:** The performance of the models over different forecast origins (PPO) Area Under Curve (AUC). 95 % confidence interval in the parenthesis

Origin	AHWM	HWDM	MHWM
0	0.52 (0.38-0.65)	0.50 (0.39-0.63)	0.50 (0.38-0.63)
1	0.46 (0.32-0.58)	0.51 (0.37-0.65)	0.51 (0.39-0.62)
2	0.50 (0.38-0.62)	0.53 (0.40-0.66)	0.51 (0.39-0.64)
3	0.46 (0.33-0.58)	0.51 (0.38-0.62)	0.55 (0.44-0.66)
4	0.46 (0.35-0.59)	0.55 (0.44-0.69)	0.60 (0.48-0.71)
5	0.60 (0.46-0.73)	0.53 (0.44-0.65)	0.56 (0.43-0.71)
6	0.56 (0.43-0.69)	0.57 (0.44-0.69)	0.58 (0.45-0.70)
7	0.60 (0.49-0.71)	0.63 (0.51-0.76)	0.56 (0.44-0.68)
8	0.70 (0.57-0.81)	0.53 (0.40-0.66)	0.69 (0.56-0.81)
9	0.70 (0.57-0.82)	0.67 (0.56-0.79)	0.58 (0.48-0.70)
10	0.67 (0.53-0.78)	0.66 (0.55-0.78)	0.66 (0.54-0.78)
11	0.65 (0.52-0.78)	0.72 (0.60-0.83)	0.69 (0.58-0.79)
12	0.68 (0.55-0.80)	0.78 (0.69-0.88)	0.76 (0.64-0.85)
13	0.74 (0.64-0.84)	0.84 (0.75-0.92)	0.80 (0.71-0.89)
14	0.81 (0.71-0.89)	0.83 (0.75-0.91)	0.84 (0.76-0.91)
15	0.87 (0.78-0.95)	0.88 (0.80-0.94)	0.90 (0.84-0.96)
16	0.82 (0.73-0.90)	0.79 (0.71-0.87)	0.85 (0.76-0.92)
17	0.75 (0.63-0.85)	0.78 (0.67-0.86)	0.75 (0.64-0.83)
18	0.74 (0.64-0.82)	0.72 (0.62-0.81)	0.73 (0.62-0.83)
19	0.60 (0.50-0.71)	0.59 (0.48-0.69)	0.57 (0.47-0.69)
20	0.56 (0.45-0.67)	0.60 (0.49-0.71)	0.56 (0.46-0.68)
21	0.61 (0.51-0.73)	0.63 (0.50-0.74)	0.59 (0.48-0.69)
22	0.59 (0.47-0.71)	0.55 (0.44-0.67)	0.58 (0.45-0.70)
23	0.55 (0.41-0.65)	0.53 (0.42-0.65)	0.51 (0.38-0.62)

While AUC is calculated over all thresholds, there are metrics, such as F1, that are calculated with a single threshold. While the models seem to have equal accuracy in terms of AUC, F1 shows clear differences: In comparison to HWDM and MHWM, AHWM performs relatively poorly when the forecast horizon is longer than four hours or when the forecast origin is between 0 a.m. and 3 p.m. (see Figs. 4 and 5 in Appendix). This is because in these cases, the sensitivity (also known as recall and true positive rate) is low, meaning that only a small fraction of crowding cases were correctly predicted in these cases.

**Table 4:** AUC matrix for AHWM. Only horizons up to $t+12$ are provided for brevity. AUC values can be calculate only if there is at least one recorded crowding event in the sample, which results in missing values marked with "-" symbol

Horizon	t+1	t+2	t+3	t+4	t+5	t+6	t+7	t+8	t+9	t+10	t+11	t+12
Origin												
0	-	-	-	-	-	-	-	-	-	-	-	-
1	-	-	-	-	-	-	-	-	-	-	-	-
2	-	-	-	-	-	-	-	-	-	-	-	-
3	-	-	-	-	-	-	-	-	-	-	-	0.33
4	-	-	-	-	-	-	-	-	-	-	1.00	0.74
5	-	-	-	-	-	-	-	-	-	1.00	0.65	0.68
6	-	-	-	-	-	-	-	-	1.00	0.94	0.76	0.56
7	-	-	-	-	-	-	-	1.00	0.96	0.81	0.62	0.52
8	-	-	-	-	-	-	1.00	0.97	0.85	0.66	0.62	0.53
9	-	-	-	-	-	1.00	0.96	0.90	0.61	0.67	0.56	0.78
10	-	-	-	-	1.00	0.91	0.78	0.51	0.49	0.58	0.70	0.62
11	-	-	-	1.00	1.00	0.85	0.60	0.64	0.73	0.84	0.73	0.52
12	-	-	1.00	0.98	0.93	0.66	0.75	0.75	0.80	0.71	0.80	1.00
13	-	0.67	0.85	0.80	0.62	0.73	0.66	0.77	0.84	1.00	1.00	-
14	1.00	0.83	0.87	0.73	0.78	0.69	0.82	0.86	0.60	1.00	-	-
15	0.92	0.76	0.73	0.94	0.74	0.89	0.75	0.74	1.00	-	-	-
16	0.90	0.82	0.95	0.82	0.92	0.83	0.80	1.00	-	-	-	-
17	0.90	0.99	0.87	0.90	0.85	0.80	1.00	-	-	-	-	-
18	0.97	0.90	0.93	0.90	0.88	1.00	-	-	-	-	-	-
19	0.93	0.97	0.83	0.94	0.00	-	-	-	-	-	-	-
20	0.93	0.96	0.92	1.00	-	-	-	-	-	-	-	-
21	1.00	1.00	1.00	-	-	-	-	-	-	-	-	-
22	0.96	1.00	-	-	-	-	-	-	-	-	-	-
23	1.00	-	-	-	-	-	-	-	-	-	-	-

## Discussion

In this study, we had three main findings. First, we showed that it is possible to build a prospective ED crowding early warning software using simple statistical forecasting models and with very limited resources. Second, we showed that even simple univariable models can provide excellent binary accuracy and potentially provide actionable information to ED stakeholders with modest computing and software requirements. Third, we showed that clinically adequete accuracy is reached with sufficient margin for preventive measures.

The PPO metric provides a simple and easily interpretable perspective into model performance. As shown in Table 3, the models had no discrimatory power at 0 a.m. but the AUC gradually increased over the course of the day, reaching 0.72 (95% CI: 0.60-0.82) at 11 a.m. and excellent level of 0.84 (95% CI: 0.74-0.91) at 1 p.m. This improvement is expected because, in this setting, the effective forecast horizon decreases as a function of increasing forecast origin. This means that the models are iteratively presented with the most recent occupancy statistics and are able to correct the prediction based on the status of the ED at prediction time. In fact, looking at the unadjusted accuracy metrics in Fig. 5, the specificity of the ETS models increases over the course of the day whereas sensitivity remains constant. Regardless, the system reached an acceptable level of accuracy as early as at 11 a.m. If the predictions were used to guide administrative decisions and since the vast majority of crowding events were observed after 6 p.m. as shown in Fig. 1b, there would have been a several hour margin for preventive measures. These measures could include calling in additional staff, obligate follow-up care facilites to accept patients over their nominal capacity, and in rare cases adjust triage on arrival.

As mentioned in the introduction, binary performance metrics have been rarely reported in previous work which makes it difficult to compare our results with that of the others. Somewhat similiar setting was used by aforementioned Hoot et al [15] in which AUC was used to determine the accuracy with which ambulance diversions would have been performed correctly up to 8 hours ahead. This is not directly comparable to Nordic ED setting, in which legislation does not warrant ambulance diversions regardless of the current crowding status. It is thus difficult to assess if the resulting threshold for crowding was similiar to that of ours. With these caveats in mind, *Hoot et al* reported $t+2$, $t+4$, $t+6$ and $t+8$ AUC values of 0.93, 0.90, 0.88, 0.85 respectively. We matched or slightly exceeded these results with respective AUC values of 0.93, 0.90, 0.90 and 0.89. It is also noteworthy, that their forecasting horizon was limited to 8 hours compared to 24 hours used here. We were able to forecast $t+24$ crowding with an AUC of 0.79.

In terms of PPH, all the models demonstrated constantly high specificity with lower and incrementally decreasing sensitivity as shown in Fig. 4. We believe this to be a result of the univariable nature of the dataset; since the models in this study did not have access to relevant exogenous variables (such as calendar variables, weather forecasts, availability of follow-up care beds etc.), the models were unable to account for sudden surges in future occupancy, which leads to false negatives and to suboptimal sensitivity. It is possible that the sensitivity of the models could be enhanced by including these variables, preferrably covering all three aspects of the Asplin’s model, and it warrants futher investigation.

Before the models are introduced to clinical practice, it is important to carefully adjust them based on clinical requirements by either raising or lowering the discriminatory threshold. This is because high number of false negatives results in repeated underresourcing and compromised quality of care. On the other hand, false positives result in overresourcing, increased operating costs and introduce the risk of developing an alarm fatigue to the end users [24]. In this article, we do not prioritize specificity or sensitivity at the cost of the other, as the optimal balance may vary based on each facility’s unique requirements and should be determined on a hospital-by-hospital basis. Technically, this could be done by weighting classes, changing the threshold, or by data sampling.

Further, it would be interesting to evaluate the performance of these models when trained on daily resolution. For example, in terms of forecasting crowding, the real targets of interest are not the hourly occupancy statistics, but the peak occupancy of the afternoon. The same models could potentially perform better, if trained with series of these daily peak occupancies, which likely demonstrate clear weekly seasonality and potential trends. Moreover, it would be likely beneficial to estimate classification techniques, such as logistic regression or support vector machines. After all, we are only marginally interested in the underlying numbers of the patients and very much interested in the binary future state of the ED and it might make sense to incorporate this logic to model cost function as well. We presume that the use of classification techniques can potentially enhance the sensitivity of the system.

The focus of this study was deliberately on building the software and integrating it into hospital information infrastructure, which was not a trivial task. For this reason, the models used were simple well-established statistical models. However, several novel machine learning based time series forecasting models have been introduced over the course of the last few years [25, 26], and these models would likely provide better accuracy in this context, especially if supplemented with relevant multivariable exogenous data.

The definition of crowding used here annotated the top 3 % of hours as crowded. Currently, in our ED, crowding protocol is triggered at even lower occupancy level of 8 %. Our threshold is thus higher than current local clinical practice and could be easily integrated to established operating culture. However, we do acknowledge that ED’s differ signicantly and these thresholds do not necessarily apply to other facilites. Further work is required to validate these findings in other centers.

## Conclusions

To conclude, we showed the ability of a prospective crowding early warning system to predict next hour crowding with an AUC of 0.94 (95% CI: 0.91-0.96) and 24 hour crowding with an AUC of 0.79 (95% CI: 0.75-0.84). We also propose a clinically oriented PPO metric and using this approach suggest that afternoon crowding can be predicted at 1 p.m. with an AUC of 0.84 (95% CI: 0.74-0.91)

Future work should 1) investigate the use of binary cost function optimally with a machine learning model, 2) investigate the accuracy of the system with stratified sample, 3) perform a pilot with the ED staff to validate the operational benefits of the system in clinical setting and 4) document the binary performance of other previously proposed multivariable ED forecasting models.

## Figures and Tables

**Fig. 3:**
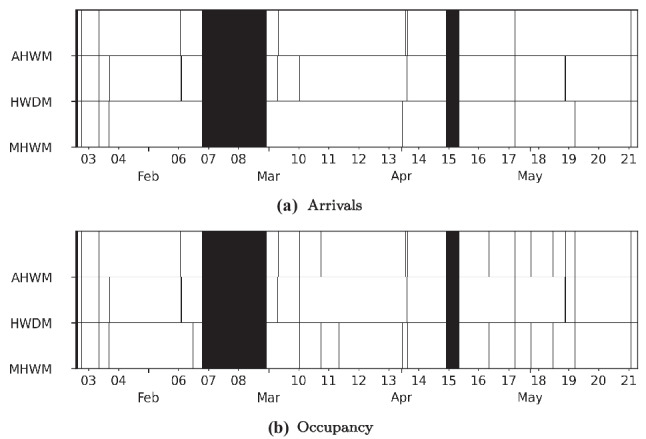
Temporal distribution of missing data, marked with black color. Two week period of missing data during weeks seven and eight are due to a credentials issue

## Appendix

### Unadjusted PPH Metrics

Unadjusted performance metrics as a function of forecast horizon are shown in Fig. 4. Overall accuracy of the models was high and nearly identical ranging between 88-97%. This was mostly explained by equally high specificity of 90-100%. AHWM had the highest one-step ahead sensitivity of 60%, but the difference compared to other models was small. The sensitivity declined as a function of increasing forecast horizon, but this effect was more prononounced with HWDM compared to AHWM and HWDM.

### Unadjusted PPO Metrics

Unadjusted performance metrics as a function of forecast origin are shown in Fig. 5. The sensitivity of AHWM was low, ranging between 0% and 20% before 1 p.m. HWDM and MHWM demonstrated identical and relatively constant sensitivity irrespective of the forecast origin with mean sensitivity of 35-38%. The specificity of AHWM was high and relatively constant with values ranging with means of 98% and 92% respectively. The specificity of HWDM and MHWM increased as a function of forecast origin from 63-65% at 1 a.m. to 94-95% at 1 p.m.
